# A Plexiform Neurofibroma Mimicking a Lipoma: A Rare Case

**DOI:** 10.7759/cureus.116553

**Published:** 2026-09-19

**Authors:** Hamza Tazi, Maria Kharbouch, Rita Ait Benhamou, Yannis Ennachachibi, Mounia El Omari

**Affiliations:** 1 Plastic and Reconstructive Surgery, Hôpital Universitaire International Cheikh Khalifa, Casablanca, MAR; 2 Pathology, Hôpital Universitaire International Cheikh Khalifa, Casablanca, MAR

**Keywords:** lipoma, lipoma mimic, neurofibroma, neurofibromatosis type 1, nf1, peripheral nerve sheath tumor, plexiform neurofibroma, pnf, soft tissue mass, von recklinghausen

## Abstract

Plexiform neurofibromas (PNFs) are rare benign tumors of the peripheral nerve sheath, typically pathognomonic of neurofibromatosis type 1, and rarely isolated. We report the case of a 48-year-old man with a medical history of polycystic renal disease with chronic renal insufficiency with dialysis who presented with a painful, slowly enlarging mass in the lower right leg. The initial clinical examination and ultrasonography misdiagnosed the mass as a lipoma. In the operating room, the lesion was found to develop under the deep fascia with malignant features. Postoperative MRI demonstrated an irregular, lobulated aspect invading multiple muscle compartments and sparing the fibular cortex. Pathology confirmed the diagnosis of PNF, with the classical spindle cells in a myxoid background and positive S-100 staining. The patient declined definitive excision. This case underscores the diagnostic difficulties of adult PNF, especially when it presents as a lipoma mimic. MRI and pathology remain the gold standard for diagnosis.

## Introduction

Plexiform neurofibroma (PNF) is a benign peripheral nerve sheath tumor that is considered pathognomonic for neurofibromatosis type 1 (NF1) [1]. It appears in childhood or adolescence and typically affects all depths of the human body where nerves are present [2]. NF1 typically manifests with cutaneous, neural, and ocular findings [3]. While PNFs are oftentimes diagnosed in syndromic NF1 cases, they sometimes present in isolated forms, creating a diagnostic challenge with more common benign tumors such as lipomas [1,4]. Correct diagnosis and early management are clinically crucial because of the 8-13% lifetime risk of malignant transformation [4].

## Case presentation

A 48-year-old male with a medical history of polycystic renal disease resulting in end-stage renal disease on hemodialysis started noticing a small mass on the lower third of his right leg, exactly superior and posterior to his lateral malleolus, at the beginning of 2023. There were no cutaneous lesions or systemic anomalies other than minimal pain and discomfort when he walked or exercised.

The patient had an initial consultation with a dermatologist, who made a preliminary diagnosis of a lipoma. Ultrasonography was requested and conducted in November 2023, which inaccurately confirmed the benign tumor. The ultrasound showed a superficial hyperechoic lesion on the lower third of his right leg. It measured 14.8 × 12.1 mm, was 5 mm from the skin surface, and appeared non-calcified and avascular (Figure 1). The patient chose not to undergo any treatment because of the non-alarming nature of the findings.

**Figure 1:**
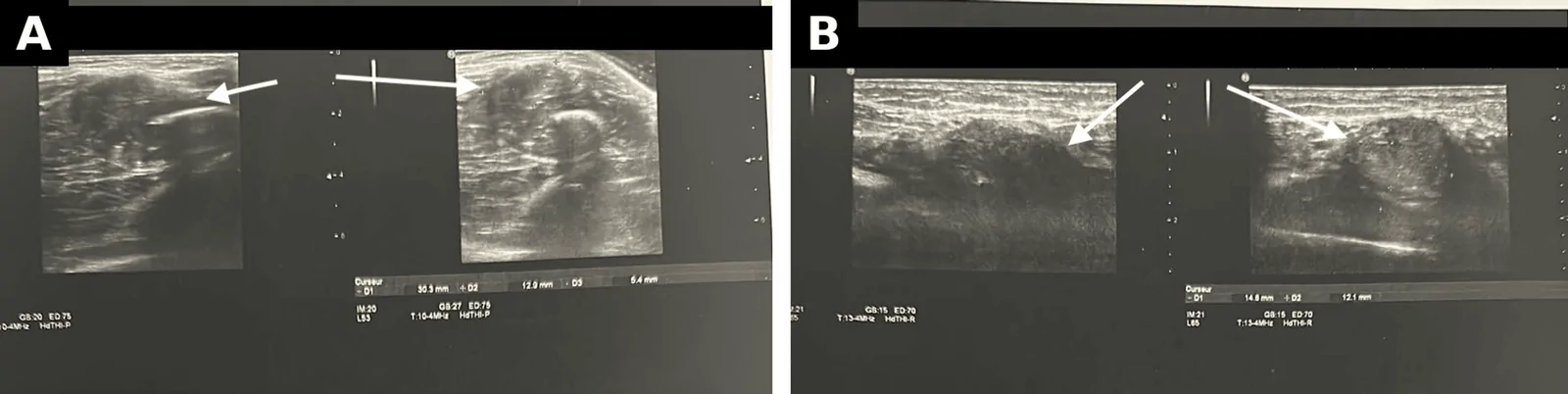
Ultrasound of the right lower leg mass at initial presentation (November 2023). (A) Transverse and longitudinal views of the mass using muscle and tendon presets; calipers show the lesion approximately 5 mm below the skin surface. (B) Longitudinal views with calipers measuring the lesion at 14.8 × 12.1 mm. The lesion appears superficial, hyperechoic, non-calcified, and avascular.

Nevertheless, at the end of 2024, his symptoms worsened, with an increase in the size of the mass and discomforting pain. He visited our plastic surgery department and was scheduled in February 2025 for excision.

Surgical and radiological findings

After the incision and starting to search for the lipoma, it quickly became noticeable that the diagnosis of lipoma was incorrect, as a mass was felt underneath the deep fascia of the leg. An incision was made through the deep fascia for further exploration. The abnormal tissue rapidly appeared and exhibited red-pale, irregular, densely fibrotic characteristics, which made us suspect a malignant neoplasm (Figure 2). A biopsy was sent to the pathology department, and an MRI was performed postoperatively.

**Figure 2:**
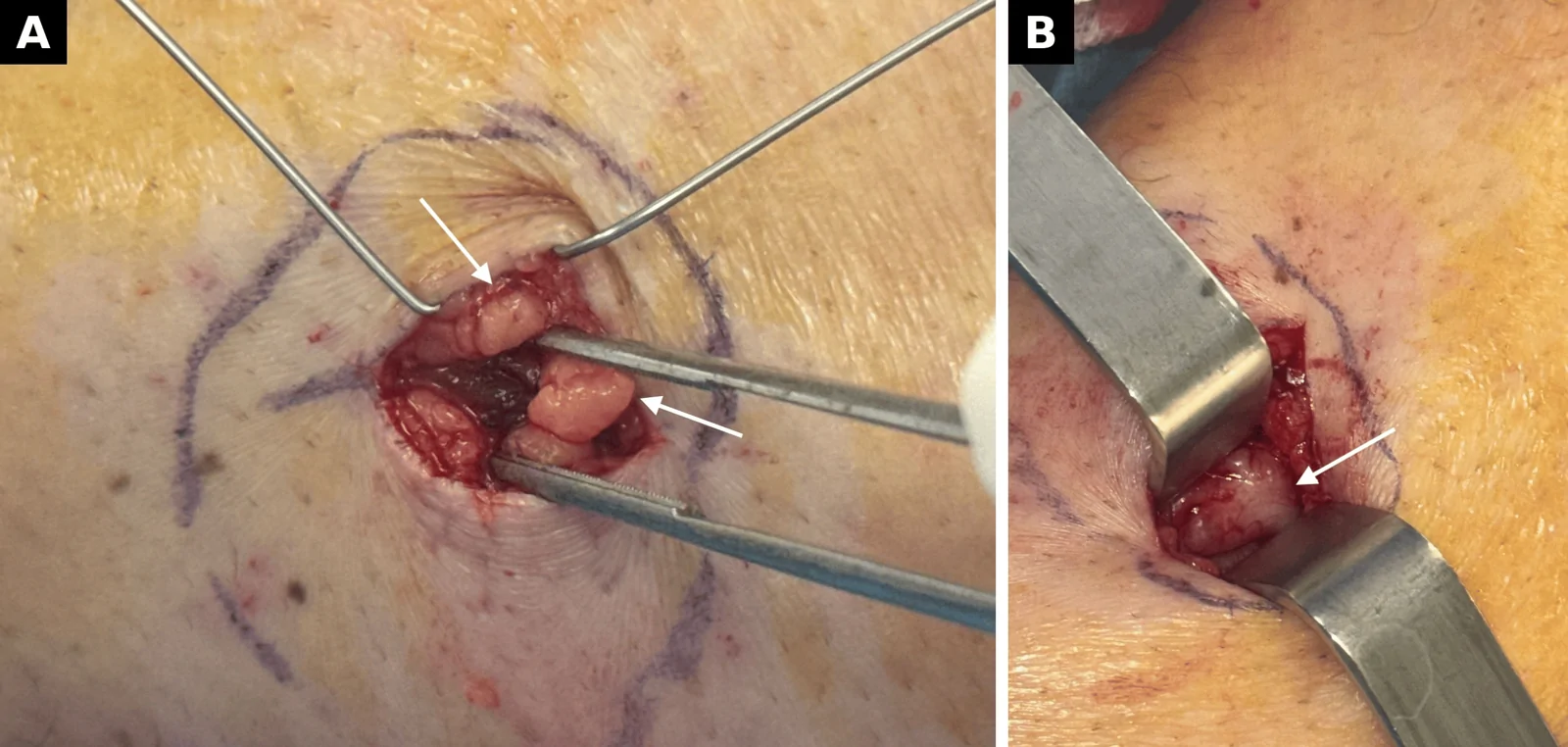
Intraoperative findings during the planned lipoma excision (February 2025). (A) Exploration through the skin incision; no lipoma was found in the subcutaneous plane, and the forceps hold the pale, irregular tissue encountered after opening the deep fascia. (B) Retractors exposing the subfascial lesion, showing red-pale, irregular, densely fibrotic tissue rather than the smooth, soft appearance of a lipoma.

The MRI showed a lesion in the posterior region of the leg, extending to the lateral side and involving the surrounding muscles. The mass was irregular, round, and lobulated with septations and necrosis. The mass was isointense on T1 and hyperintense on short tau inversion recovery sequences with enhancement after gadolinium administration. It measured 35 mm (anteroposterior), 28 mm (lateral), and 49 mm (height), and encapsulated the fibula on its posterior circumference (Figures 3, 4). The radiology department requested a CT scan to evaluate cortical infiltration, which was negative.

**Figure 3:**
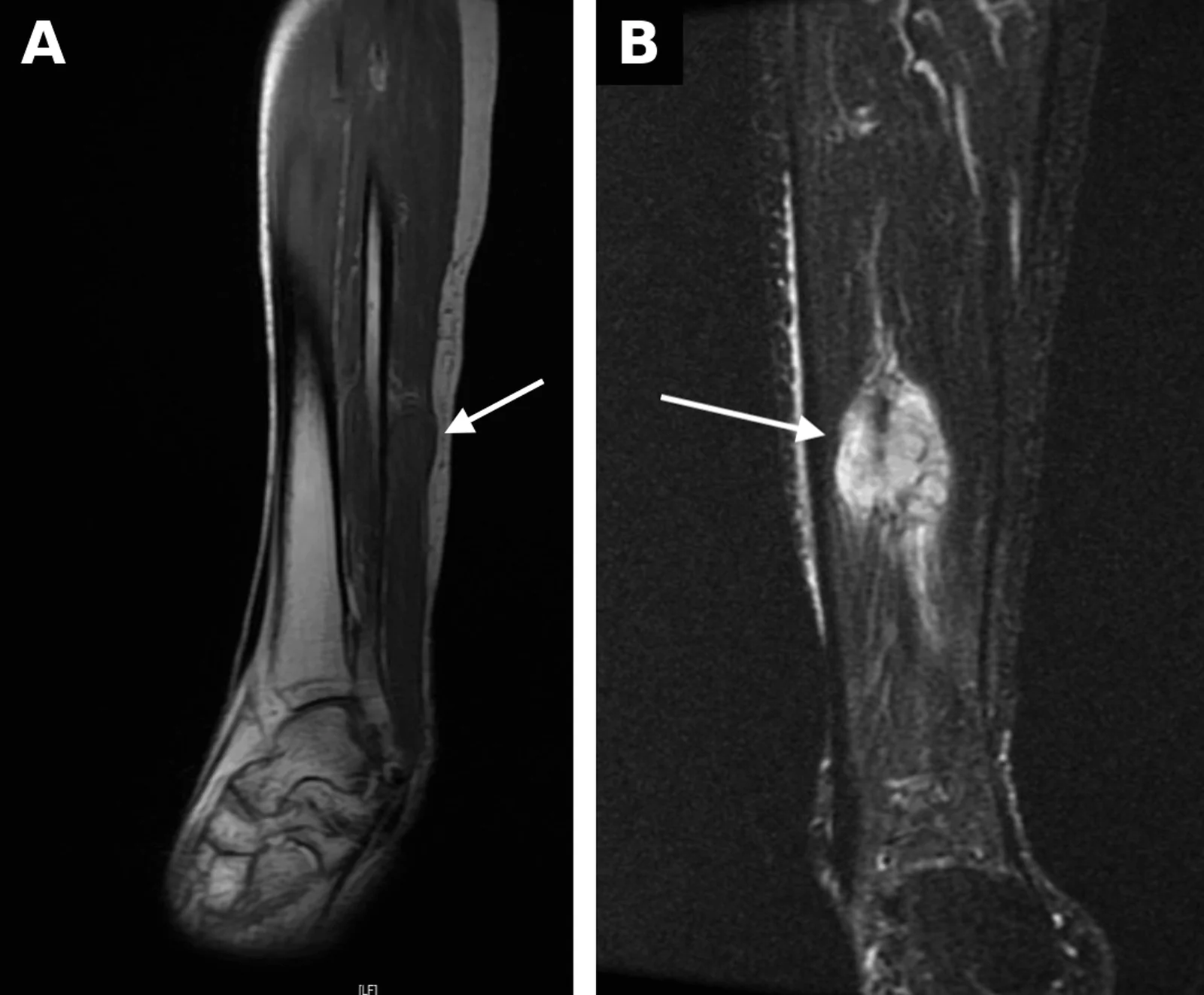
Postoperative saggital and coronal MRI of the right lower leg showing the craniocaudal extent of the mass and its relationship to the fibula. (A) Sagittal T1-weighted image: the mass is isointense to muscle, with preserved bright signal of the subcutaneous fat and bone marrow. (B) Coronal STIR image: the mass is markedly hyperintense, showing its craniocaudal extent along the posterolateral leg. STIR: short tau inversion recovery

**Figure 4:**
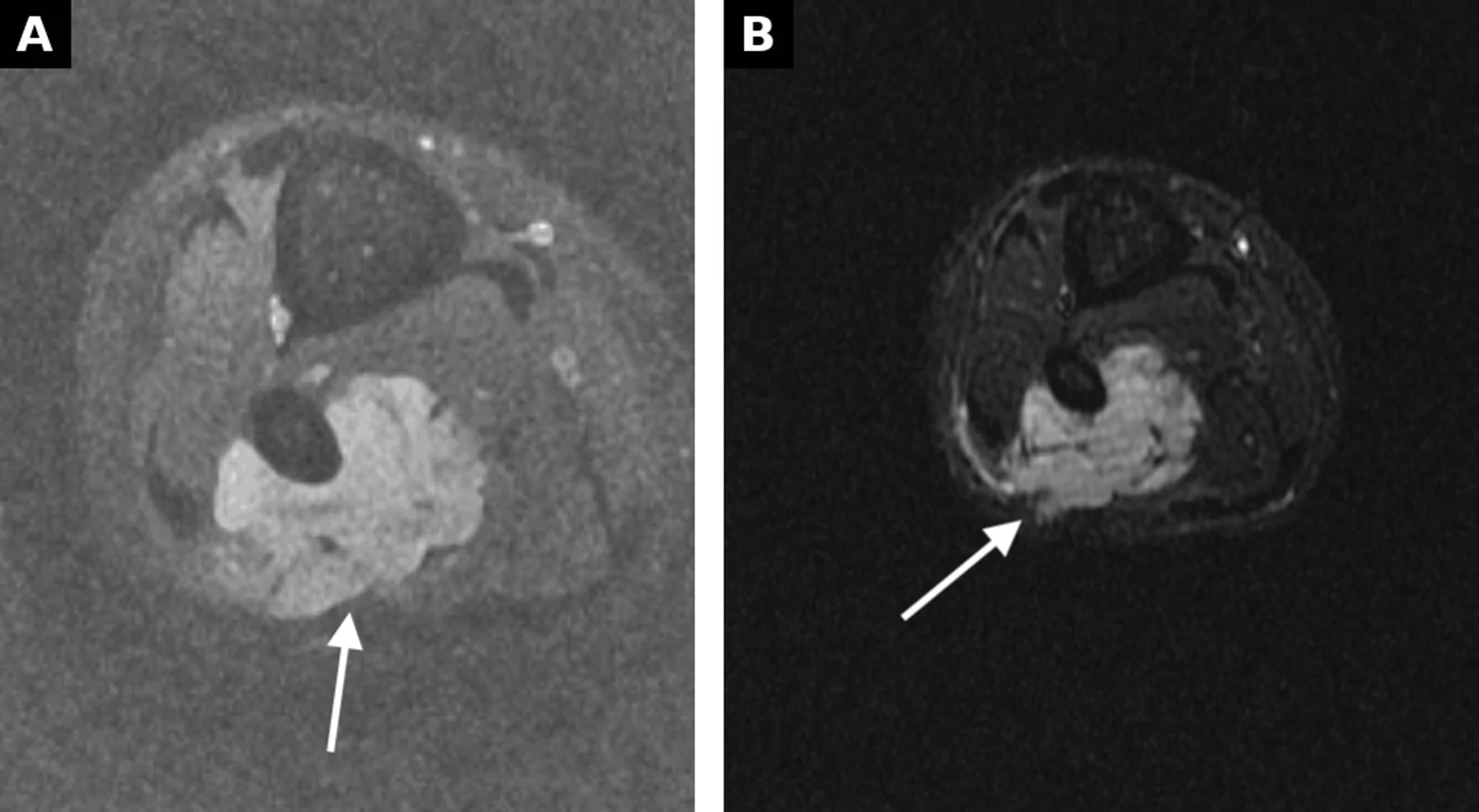
Postoperative axial MRI of the right lower leg showing the lobulated subfascial mass involving the posterior and lateral compartments. (A) Axial contrast-enhanced fat-saturated T1-weighted image: lobulated, enhancing mass in the posterior and lateral compartments, wrapping around the posterior circumference of the fibula. (B) Axial STIR image: the same mass is hyperintense with septations, without cortical involvement of the fibula. STIR: short tau inversion recovery

Histopathological findings

Microscopic analysis revealed tortuous, plexiform bundles of spindle-shaped cells corresponding to enlarged nerve branches on a myxoid background. The tumor cells, with elongated nuclei and pointed tips, showed very slight cytonuclear atypia and rare mitotic figures. A few adipocyte clusters were embedded in this proliferation (Figures 5, 6).

**Figure 5:**
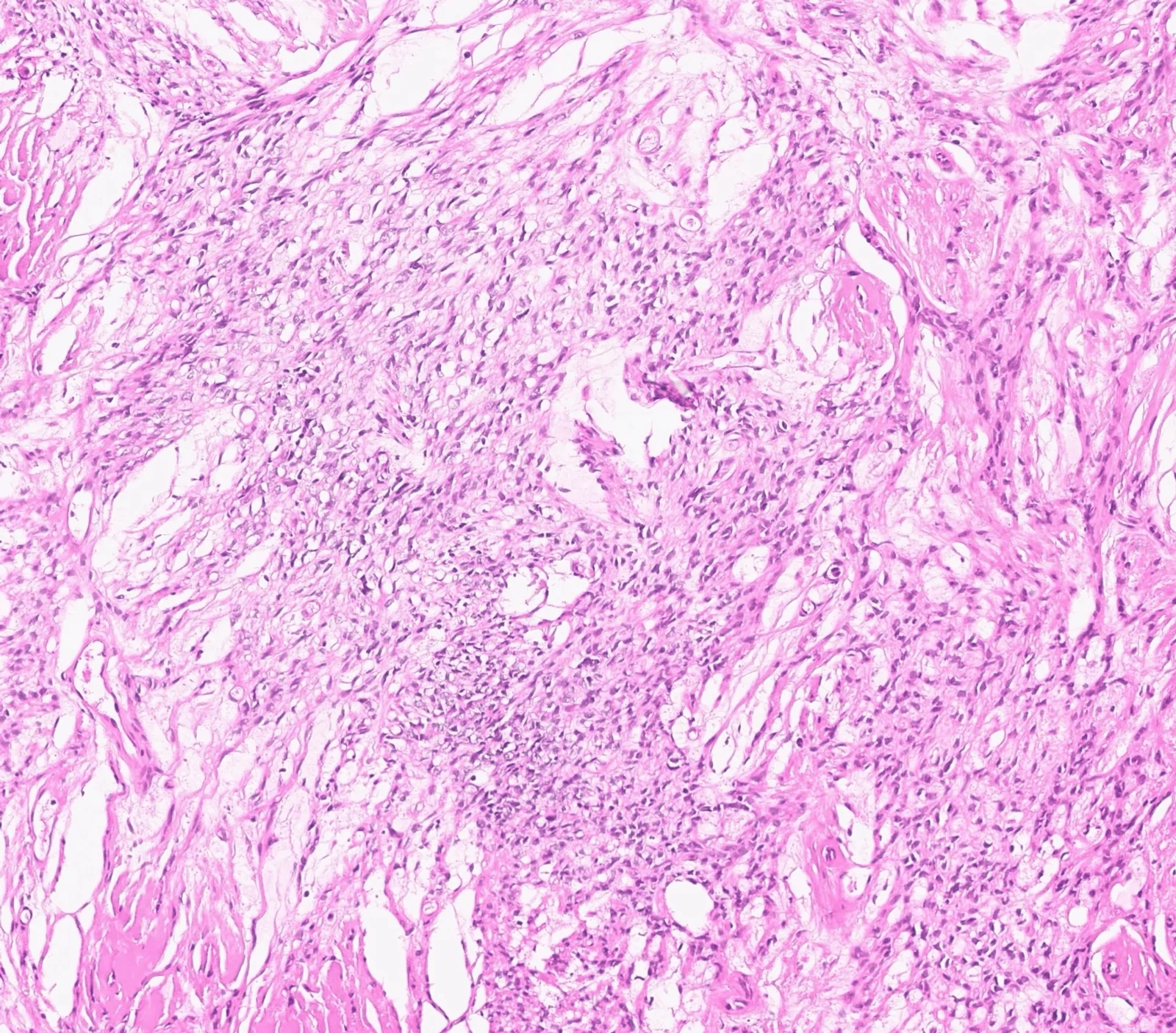
High-power view showing elongated nuclei with pointed ends, without cytonuclear atypia (hematoxylin and eosin, 20×).

**Figure 6:**
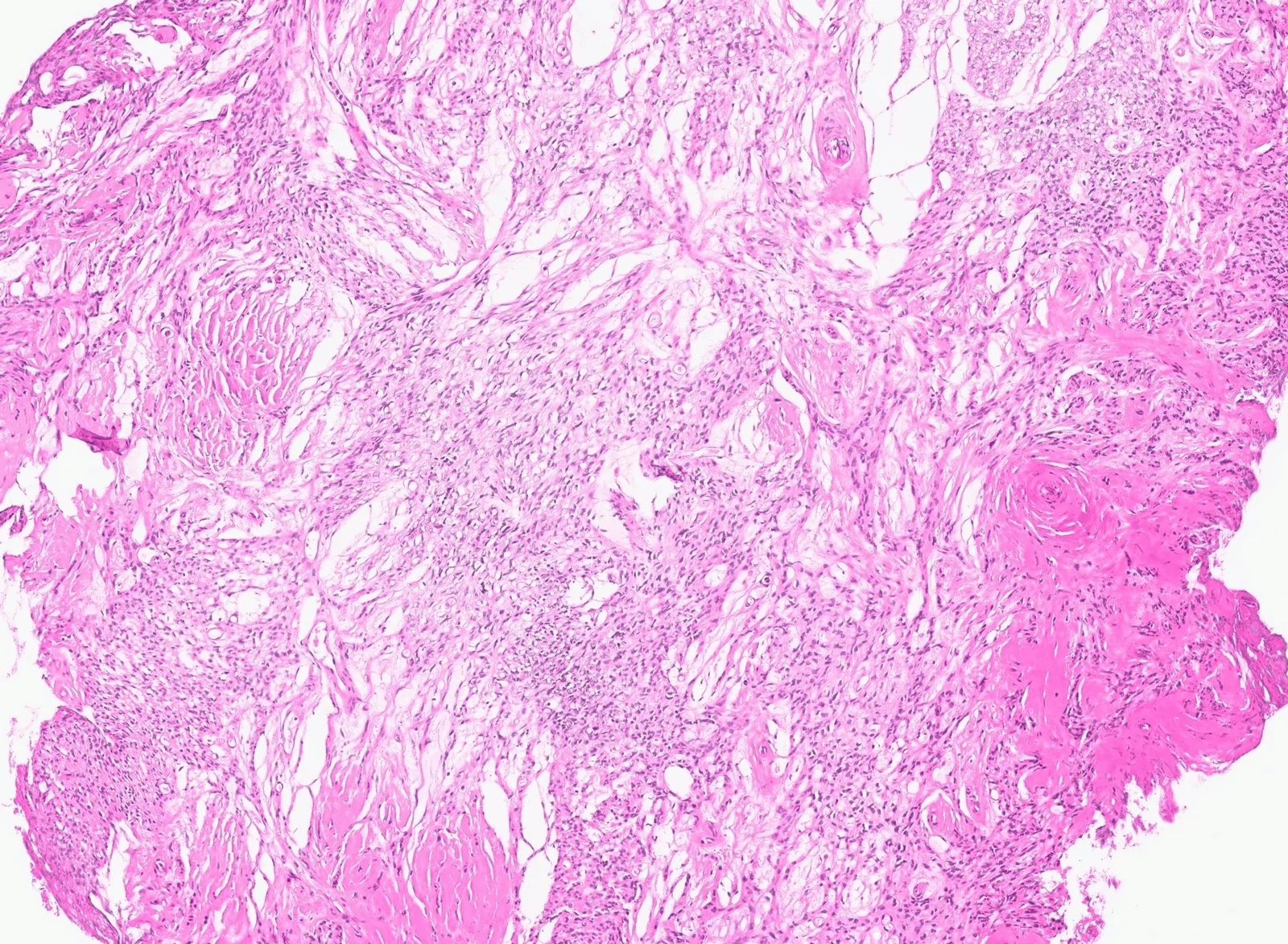
Low-power view showing tortuous pattern with nest of spindle-shaped cells (hematoxylin and eosin, 10×).

Immunohistochemical complement revealed diffuse positivity of tumor cells by S-100, as well as weak and heterogeneous labeling of tumor cells by EMA, leading to the diagnosis of PNF (Figure 7).

**Figure 7:**
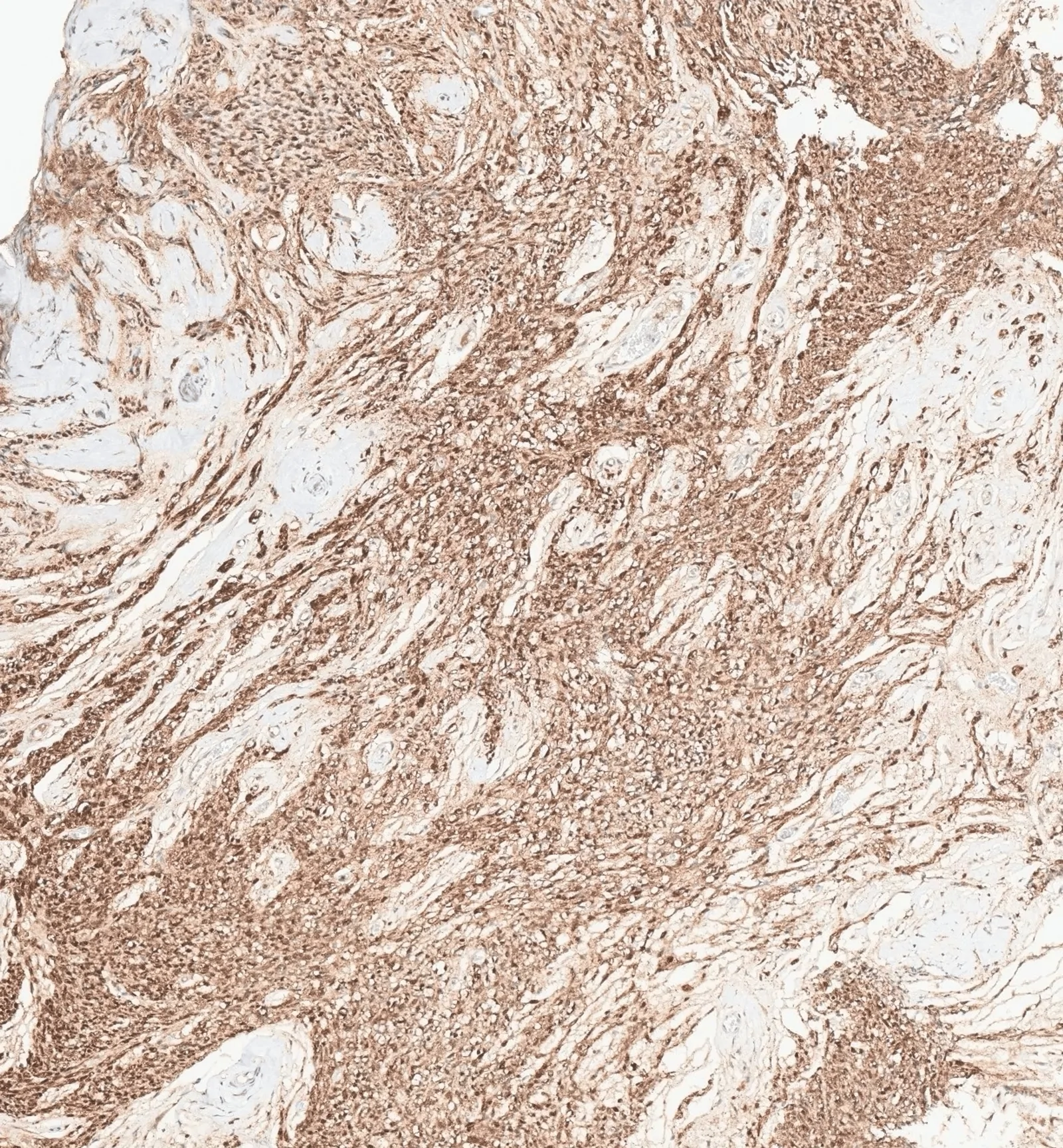
Positive S-100 immunostaining showing characteristic large plexiform bundles.

Unfortunately, our patient, after thorough explanations, refused to undergo excision surgery.

## Discussion

PNF is classified as a peripheral nerve sheath tumor pathognomonic of NF1 [1]. In clinical presentations of isolated PNF without other signs of NF1, it can be misdiagnosed as a lipoma, especially in cases where it is in the subcutis and enlarges slowly with no symptoms [1,4]. In a specific case, an isolated PNF was diagnosed as a lipoma, and only after surgery was NF1 revealed [1]. In our case, the tumor was misdiagnosed as a lipoma based on the physical examination and ultrasonography findings, emphasizing how PNF could simulate more common benign tumors. Thorough evaluation emphasizing family history and other skin lesions is crucial when clinical evaluation and imaging become atypical [1,4].

Ultrasound often leads to incorrect diagnoses of PNFs. They appear hyperechoic and avascular without defining features, closely mimicking lipomas when superficial [2]. High-resolution scanning sometimes shows nerve continuity, signaling neurogenic origin. MRI is considered the gold standard for diagnosing PNFs. Typical signs are multilobulated, elongated masses along nerve pathways. On T2 imaging, the target sign, which is central hypointensity with peripheral hyperintensity, is characteristic of PNF but not always present [2,3]. In our case, MRI demonstrated an irregular, enhanced, separated mass with muscle infiltration, raising concerns for a possible sarcoma.

CT is useful to assess bone infiltration and calcifications. The CT in our case was able to confirm the absence of cortical invasion of the fibula despite it being encapsulated, thus favoring a benign tumor [3]. Positron emission tomography scans can differentiate malignant peripheral nerve sheath tumors (MPNSTs) from PNFs, as the malignant form has higher metabolic activity [2].

Intraoperative findings usually reveal irregular, diffuse, infiltrative fibrotic tissue in contrast with the smooth and soft appearance of a lipoma [3,4]. In our case, the deep fascia incision uncovered a mass with malignant characteristics, indicating biopsy and MRI before removal. Features such as firmness, poor demarcation, stiffness, and hard consistency should raise suspicion. Complete excision is generally not simple, as PNF is generally vascular and encompasses other structures, notably nerves, muscles, and bone [4].

Pathology is key to the definitive diagnosis. Microscopically, PNFs show tortuous bundles of spindle-shaped Schwann cells with a myxoid background, with minimal atypia and low mitotic activity [5]. Immunohistochemically, PNFs are positive for S-100 (indicating Schwann cell origin) and often show positive EMA on perineurial cells [5,6]. Fibroblastic stromal cells show CD34 staining, common in PNF and rare in schwannomas. The combination of these markers rules out MPNST and spindle cell lipomas. In our case, PNF was confirmed through positive S-100 and EMA staining [5].

In adults specifically, an evaluation of NF1 should be prompted after diagnosis of PNF, regardless of the presence or absence of symptoms [1]. Genetic counseling should be started. Surgical treatment is the main therapeutic solution, and intraoperatively, a decision should be made between complete excision and nerve preservation [3,4]. Incomplete resections occur due to the infiltrative growth patterns of the tumor; however, debulking has been shown to relieve symptoms [3]. There is an 8-13% lifetime risk of conversion of PNF into a malignant form, prompting long-term surveillance [4]. Any rapid growth, pain, or new neurological symptom should prompt immediate complete reevaluation. Nowadays, MEK inhibitors (selumetinib) show favorable results for recurrent or inoperable PNFs, especially in pediatric patients [4].

## Conclusions

Adult PNF poses diagnostic, surgical, and long-term management challenges. This case demonstrated how a PNF could be misread as a lipoma and overlooked for years until recognized in the operating room and the pathology laboratory. A multidisciplinary approach is crucial for diagnosis and optimal treatment. With new emerging therapies, results can be favorable even in more complex cases. Early treatment is frequently necessary to prevent injury to nearby anatomical structures and transformation into a malignant tumor.
